# Supplementary material for: Activity data from wearables as an indicator of functional capacity in patients with cardiovascular disease
Source: PLoS One. 2021 Mar 24;16(3):e0247834. doi: 10.1371/journal.pone.0247834 (PMC7990307; doi:10.1371/journal.pone.0247834)
Supplement: S1 Appendix — (DOCX) [file pone.0247834.s001.docx]

S1 Appendix.

Activity data from wearables as an indicator of functional capacity in patients with cardiovascular disease

Neil Rens^1,2¶^*, Neil Gandhi^1,2¶^, Jonathan Mak^1&^, Jeddeo Paul^1,2&^, Drew Bent^2^, Stephanie Liu^2^, Dasha Savage^1^, Helle Nielsen-Bowles^1^, Doran Triggs^1^, Ghausia Ata^1^, Julia Talgo^1^, Santiago Gutierrez^1^, Oliver Aalami^1^

^1^Department of Surgery, Division of Vascular Surgery, Stanford University, Stanford, California, USA

^2^Graduate School of Business, Stanford University, Stanford, California, USA

^#a^Current Address: 650 Jane Stanford Way, W118, Stanford, CA 94305

*Corresponding author

Email: neilerens@gmail.com

^¶^These authors contributed equally to this work.

^&^These authors also contributed equally to this work.

**Activity graph**

The activity graph is a visual representation of a patient’s daily step count throughout the 6 month study period (Fig S1). We developed this graph to be easily interpreted and acted upon by a clinician. We observed considerable day-to-day variation in daily steps counts (blue line), so we used a moving average to generate trendlines (green and orange) that capture changes in functional status without the noise of the raw step counts. Although this graphical representation was not included in our analysis, it helped us understand a patient’s trajectory and would likely be helpful to clinicians who are attempting to interpret remote activity data in real time.

**Fig S1. Activity graph.** The purple vertical line denotes an intervention (surgical or percutaneous, depending on the patient). The blue line represents raw daily total steps walked. The light orange line is a 14 day exponential moving average taken twice on the total steps, putting more weight on near values. The light green line is the simple 14 day moving average taken twice, putting equal weight on all values. Both moving averages help elucidate the trends in activity, but the exponential moving average (orange) is also able to capture detail about day-to-day changes in activity.


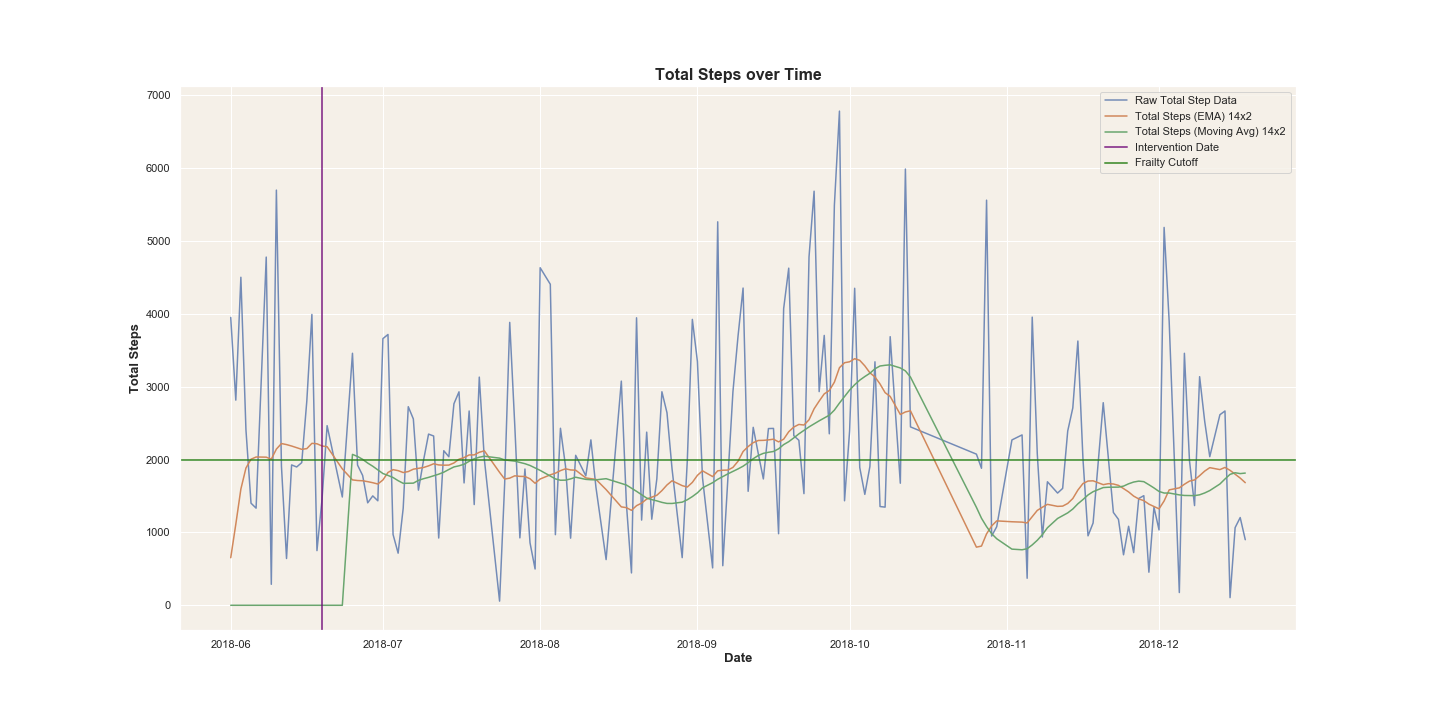


**Using smartphone data from home to predict clinic 6MWT performance**

**Feature engineering**

In order to predict clinic 6MWT performance from home data, we created various features to test in the logistic regression model. The model schematic can be seen in Fig S2. Note: MSWS refers to maximum steps without stopping, which is the highest number of steps a participant walked continuously on a given day.

**From the home-based 6MWT, the features were:**

1. Total steps, from the **phone**
2. Total steps, from the **watch**
3. Total steps, from the **watch or phone, whichever was higher**
4. Log of feature 1
5. Log of feature 2
6. Log of feature 3

**From the passive activity data, the features were:**

1. Average of daily steps over the preceding 7 days, from the **phone**
2. Average of daily steps over the preceding 7 days, from the **watch**
3. Average of daily steps over the preceding 7 days, from the **watch or phone, whichever was higher**
4. Highest daily step count over the preceding 7 days, from the **phone**
5. Highest daily step count over the preceding 7 days, from the **watch**
6. Highest daily step count over the preceding 7 days, from the **watch or phone, whichever was higher**
7. Log of feature 1
8. Log of feature 2
9. Log of feature 3
10. Log of feature 4
11. Log of feature 5
12. Log of feature 6
13. Average of MSWS over the preceding 7 days, from the **phone**
14. Average of MSWS over the preceding 7 days, from the **watch**
15. Average of MSWS over the preceding 7 days, from the **watch or phone, whichever was higher**
16. Highest MSWS over the preceding 7 days, from the **phone**
17. Highest MSWS over the preceding 7 days, from the **watch**
18. Highest MSWS over the preceding 7 days, from the **watch or phone, whichever was higher**
19. Log of feature 13
20. Log of feature 14
21. Log of feature 15
22. Log of feature 16
23. Log of feature 17
24. Log of feature 18

**Fig S2. Predictive model schematic.**


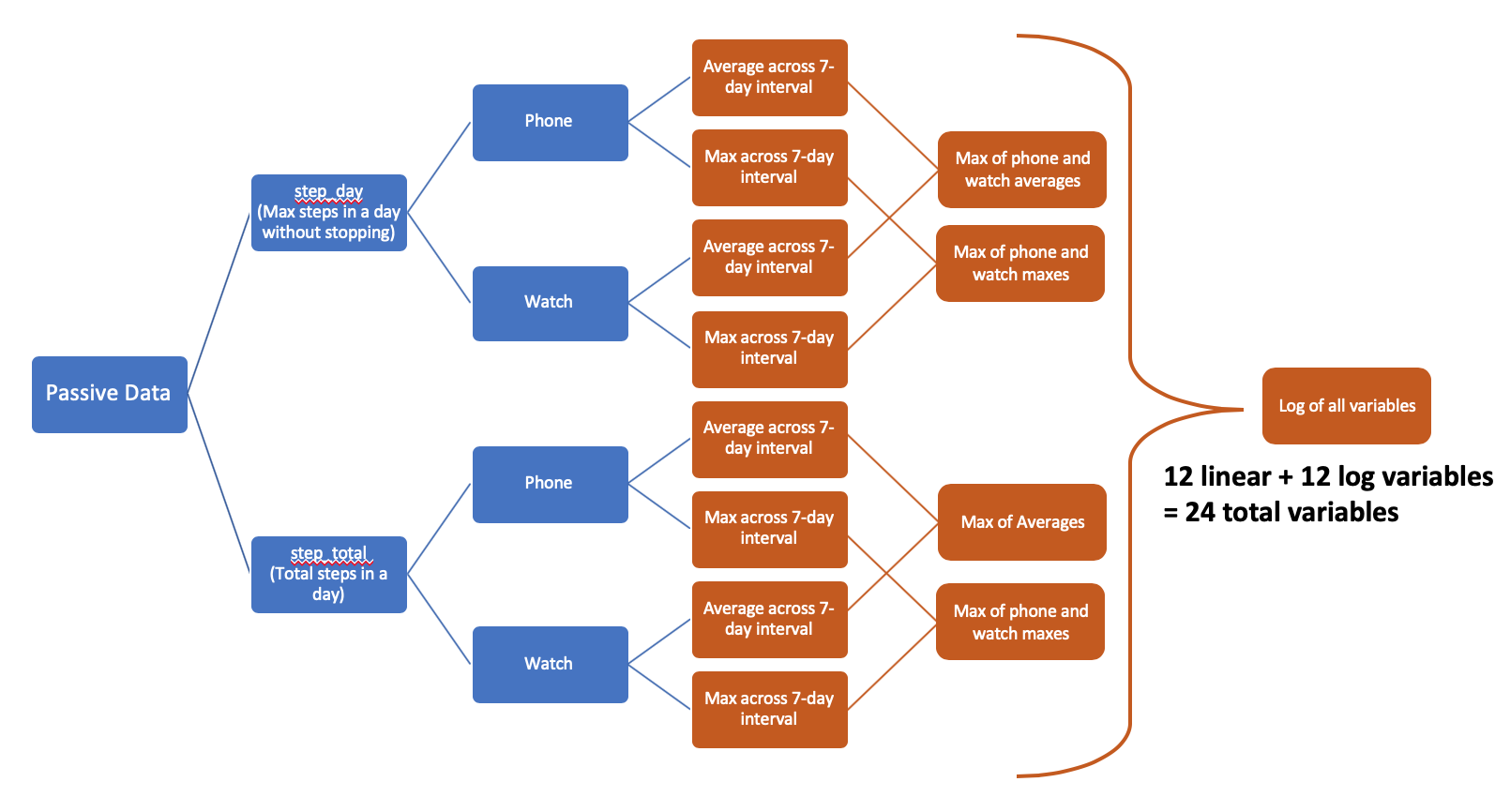


**Bootstrapping**

Although the variables included in our predictive models were determined via a five-fold cross validation repeated ten times, testing of the model was only performed on a single, randomly chosen testing set, which is prone to variability. We therefore utilized a bootstrapping approach to determine a 95% confidence interval for each model’s true AUC. Using bootstrapping (described [here](https://journals.sagepub.com/doi/pdf/10.1177/0962280216680242) and [here](https://books.google.com/books/about/An_Introduction_to_the_Bootstrap.html?id=gLlpIUxRntoC)), we sampled with replacement from our original testing dataset to create 200 new training datasets, which were validated against 1000 testing datasets created in a similar fashion. Mean AUC was calculated across the 200 trials run against a single bootstrapped testing dataset, and a 95% confidence interval was constructed from the resulting 1000 mean AUCs. Results are shown in Fig S3. Note: for bootstrapping, our “frailty” outcome was defined as <500 steps, which corresponds to <300m per Fig 1.

**Fig S3. Bootstrapped AUCs for baseline and experimental models predicting in-clinic walk test performance.** Bootstrapped Mean AUC in baseline model = 0.612, 95% CI = [0.310 0.848]. Bootstrapped Mean AUC in experimental model = 0.747, 95% CI = [0.538 0.884].

*
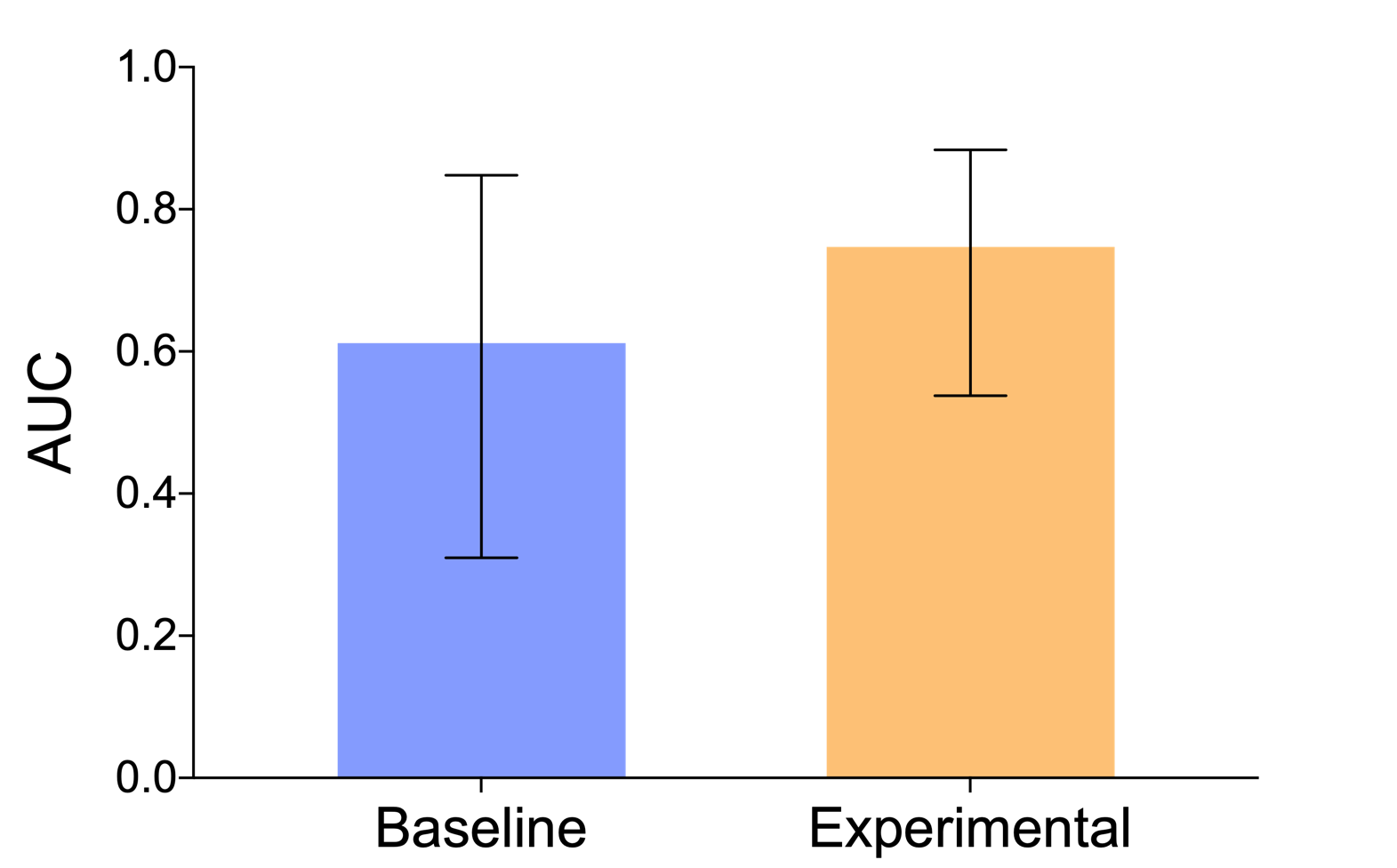
*

**Participant adherence**

Adherence measures are based on all participants across all study days (Table S1). Days Connected corresponds to how many days the patient’s device was turned on and able to collect/transmit data. Days Active corresponds to how many days the patient’s device actually registered steps. A day where no steps were registered could be due to the patient not keeping their phone on their body or due to hardware issues. Days in Study corresponds to how many days the patient was enrolled in the study.

**Table S1: Participant adherence.**

|  | Either (%) | Phone (%) | Watch (%) |
| --- | --- | --- | --- |
| Days Active / Days Connected | 93.1 | 87.5 | 95.8 |
| Days Active / Days In Study | 73.8 | 63.5 | 66.2 |
| Days Connected / Days In Study | 77.9 | 70.1 | 67.9 |
