# Supplementary material for: Activity data from wearables as an indicator of functional capacity in patients with cardiovascular disease
Source: PLoS One. 2021 Mar 24;16(3):e0247834. doi: 10.1371/journal.pone.0247834 (PMC7990307; doi:10.1371/journal.pone.0247834)
Supplement: Attachment — Submitted filename: Resposne to Reviewers.pdf [file pone.0247834.s003.pdf]

Stanford MEDICINE | Vascular & Endovascular Surgery

Neil Rens, MSc
(619) 245-8989
neilerens@gmail.com

Alway Building, M121
300 Pasteur Drive
Stanford, CA 94305-5639

Dear Reviewers,

February 1, 2021

Thank you for your thoughtful reading of our manuscript entitled *Activity data from wearables as an indicator of functional capacity in patients with cardiovascular disease*. You highlighted some important considerations that we address below point-by-point. Additionally we are now providing the de-identified data as supplementary information and have updated our financial disclosure to clarify further about a grant we received from Apple, Inc.

We appreciate your reconsideration of this manuscript for publication.

Sincerely,

Neil Rens, MSc
On behalf of all authors

Reviewer #1: Although the passive data were continuously recorded, it is not possible to exclude that the participation to the study protocol acted itself as a "Motivator", inducing patients to be somehow more active than usual.
Thank you for raising this important insight. We have included a few sentences in the discussion (line 268) addressing the fact that the study protocol itself may have been an activity motivator and therefore patient activity outside of a study protocol may differ:

> Additionally, the participants may have been subject to the Hawthorne effect such that their awareness of being enrolled and monitored may have induced more activity than was their baseline. However, implementation of VascTrac monitoring outside of a research study could still induce this effect, and regardless of the patients' level of activity our results suggest that the VascTrac system is capable of accurately capturing passive activity data.

Reviewer #2: Future question – validate longitudinal outcomes with activity data

We wholeheartedly agree that future studies should assess longitudinal outcomes. We acknowledged that we did not have enough interventions in our study cohort to apply regression analysis to assess outcomes. We have also added the following language to the discussion of study limitations (line 258):

> We also only collected ~5 months of post-surgery data, but future studies with longer time horizons could capture longitudinal outcomes, such as 1, 2, 3 or 5-year morbidity and mortality data. Longitudinal data are also important to assess sustained patient engagement, which is known to decrease over time especially when there are fewer in-person visits [23].

Reviewer #2: Dropout rate – suggests a possible current limitation of remote data as a reliable clinical tool in all clinical settings – this may be a barrier to reliance on this emerging technology for certain potentially marginalized patient cohorts
You raise an important point about the potential for widespread adoption of remote monitoring. We have added additional discussion about the dropout rate in our study population and how that dropout rate may differ for other marginalized patient populations (line 248):

> Dropout rate has previously been a barrier to the adoption of remote data as a reliable clinical tool. While our study suggests that remote monitoring may be reliable among veterans, it is possible that remote monitoring may not be as effective for other marginalized patient populations, particularly those with intermittent access to electricity and cellular service and those with high degrees of medical mistrust. Accessibility remains a major consideration for widespread implementation.

Reviewer #2: Would be interesting to assess "frailty" assessment with other indicators of comorbidity and or a total burden of comorbidities
Reviewer #2: Please comment on the determination of "frailty" and resource allocation in the context of care and or prognostic information
Thank you for highlighting this question about the clinical utility of frailty. We have added a paragraph to the discussion to explain the clinical context for frailty and have added a citation for various other frailty measures. While other non-activity based measures of frailty use other indicators of comorbidity, our study did not have access to this data and therefore we could not compare frailty as predicted by remote monitoring these other frailty measures (line 216).

> Frailty has been shown to be an independent risk factor for adverse outcomes across surgical specialties [22]. Many tools such as the modified Frailty Index, Memorial Sloan Kettering–Frailty Index, geriatric assessment, and the Risk Analysis Index have been developed as more objective measures to supplant the "eyeball test." When considering

> an elective procedure, patients that are deemed “frail” would ideally be enrolled in a “prehabilitation” program to optimize their nutrition and functional status. In urgent cases, patients deemed “frail” would be advised to have lower risk, less invasive procedures. A better understanding of surgical risk enhances the shared decision-making required to respect patient preferences and quality-of-life considerations. Passive activity data collection on a smartphone could be viewed as “activity as a vital sign” and an excellent proxy for frailty with excellent negative predictive values.

Reviewer #2: It is important to comment on the long term prospects for sustained patient engagement and use when incorporating novel digital technology and data collection in patient care (home, outpatient, inpatient settings)
While we agree this is an important topic to discuss, given the 6-month time period of this study, we do not feel we have the data to extrapolate about sustaining patient engagement beyond 6 months. However, we have added a reference to a relevant study on patient engagement during a home-based exercise intervention monitored with a wearable (line 258):

> We also only collected ~5 months of post-surgery data, but future studies with longer time horizons could capture longitudinal outcomes, such as 1, 2, 3 or 5-year morbidity and mortality data. Longitudinal data are also important to assess sustained patient engagement, which is known to decrease over time especially when there are fewer in-person visits [23].
